# Detection of circulating epithelial cells in the blood of patients with breast cancer: comparison of three techniques

**DOI:** 10.1038/sj.bjc.6602418

**Published:** 2005-02-15

**Authors:** A E Ring, L Zabaglo, M G Ormerod, I E Smith, M Dowsett

**Affiliations:** 1 Academic Department of Biochemistry, Royal Marsden Hospital, Fulham Road, London SW3 6JJ, UK; 2 Breast Unit, Royal Marsden Hospital, Fulham Road, London SW3 6JJ, UK

**Keywords:** breast cancer, micrometastases, circulating tumour cells

## Abstract

This study compares the sensitivities and specificities of three techniques for the detection of circulating epithelial cells in the blood of patients with breast cancer. The number of circulating epithelial cells present in the blood of 40 patients with metastatic breast cancer and 20 healthy volunteers was determined by: immunomagnetic separation (IMS) and laser scanning cytometry (LSC), cell filtration and LSC and a multimarker real-time RT–PCR assay. Numbers of cytokeratin-positive cells identified and expression of three PCR markers were significantly higher in the blood of patients with breast cancer than in healthy volunteers. Using the upper 95% confidence interval of cells detected in controls to determine positive patient samples: 30% of patients with metastatic breast cancer were positive following cell filtration, 48% following IMS, and 60, 45 and 35% using real-time RT–PCR for cytokeratin 19, mammaglobin and prolactin-inducible peptide. Samples were significantly more likely to be positive for at least one PCR marker than by cell filtration (83 *vs* 30%, *P*<0.001) or IMS (83 *vs* 48%, *P*<0.001).The use of a multimarker real-time RT–PCR assay was therefore found to be the most sensitive technique for the detection of circulating epithelial cells in the blood of patients with breast cancer.

Over recent years, it has become apparent that circulating epithelial cells, assumed to represent metastatic tumour cells, can be detected in the blood of many patients with breast cancer (Ring *et al*, 2004). The detection of such cells could have significant clinical utility in risk stratification in early breast cancer, in early detection of relapse and in monitoring response to treatment. Cytometric techniques based on immunohistochemical analyses and nucleic acid-based approaches to cell detection have been described (Racila *et al*, 1998; Lambrechts *et al*, 1999; Smith *et al*, 2000; Aerts *et al*, 2001; Stathopoulou *et al*, 2002; Witzig *et al*, 2002). However, there is considerable variability in the reported sensitivities and specificities of existing techniques with putative carcinoma cells reported to be present in between 0 and 100% of blood samples from patients with metastatic breast cancer (Ring *et al*, 2004). Unfortunately, the design of published studies makes it difficult to compare directly the performance of current techniques and therefore to ascertain the optimal approach.

In the current study, we directly compare three techniques for the detection of circulating epithelial cells in the blood of patients with breast cancer and a healthy volunteer control population. The first technique combines positive immunomagnetic separation (IMS) with laser scanning cytometry (LSC), techniques that have both previously been applied to circulating epithelial cell detection (Brandt *et al*, 1998; Pachmann *et al*, 2001; Witzig *et al*, 2002). The second technique uses a novel approach to enrichment based on cell filtration as described by Vona *et al* (2000), and previously used in our own studies in combination with LSC (Zabaglo *et al*, 2003). The final method of analysis is a multiple marker real-time RT–PCR assay. The PCR assay was developed to detect cytokeratin 19 (CK19), mammaglobin and prolactin-inducible peptide (PIP) (Clark *et al*, 1999), which are expressed in breast epithelial cells but at low levels in normal blood components. CK19 was chosen as it is expressed in the majority of breast cancers (Bartek *et al*, 1986; Moll, 1994), and has been extensively used in studies in this area (Slade *et al*, 1999; Smith *et al*, 2000; Aerts *et al*, 2001; Stathopoulou *et al*, 2002, 2003). Mammaglobin gene expression has previously been used to detect circulating breast cancer micrometastases with no false positives in control populations (Zach *et al*, 1999; Silva *et al*, 2002). In a recent study, mammaglobins, and the third marker PIP, were found to be the most sensitive and specific markers for the detection of micrometastatic breast cancer in axillary lymph nodes (Mitas *et al*, 2003).

## MATERIALS AND METHODS

### Clinical samples

Ethical approval was granted for this study by the Royal Marsden Hospital Local Research and Ethics Committee, and written informed consent was obtained. Patients were eligible if they had metastatic breast cancer (stage IV according to AJCC criteria (Singletary *et al*, 2002)) and were not receiving cytotoxic chemotherapy at the time of enrolment (endocrine and bisphosphonate therapy were permitted). Healthy volunteers were selected from the staff of the Academic Department of Biochemistry and the Royal Marsden Hospital Breast Unit. One 18 ml blood sample was taken from each subject via a peripheral vein. Venepuncture was with a 21- or 23-G needle, and the first 5 ml were discarded to minimise contamination by skin epithelial cells. Samples were taken into EDTA tubes (Greiner Bio-One Ltd, Stonehouse, UK) and processed within 30 min of venesection.

All analyses were conducted blind of the patient/control status of the samples.

Each sample was split into 6 ml aliquots for analysis by one of the following three techniques:

*IMS followed by LSC*: The 6 ml blood sample was subjected to density gradient enrichment over an equal volume of Histopaque-1077 (Sigma-Aldrich Company Ltd, Poole, UK). The protocol used and PBS washes were according to the manufacturer's instructions. The resulting cell pellet was resuspended in 100 *μ*l of PBS and incubated with mouse BerEP4-FITC (20 *μ*g ml^−1^) antibody (Dako, Cambridge, UK) for 10–15 min at room temperature. Two washes were performed in IMS buffer (Ca^2+^- and Mg^2+^-free PBS, 0.5% bovine serum albumin and 2 mM EDTA) prior to incubation with goat anti-mouse IgG Microbeads (Miltenyi-Biotec Ltd, Surrey, UK) for 15 min at 4°C. After further washes in buffer, the cells were subjected to magnetic separation using a MiniMACS separation column (Miltenyi-Biotec Ltd, Surrey, UK). The positive fraction was centrifuged and resuspended in PBS and a cytospin prepared using a Hettich Universal 32R centrifuge (Hettich-Zentrifugen, Germany). The cytospin spot deposited on a microscope slide was fixed in 100% methanol for 10 min and air-dried.

For cytokeratin staining slides were rehydrated in PBS followed by TBP (0.5% Triton X-100 and 0.5% bovine serum albumin in PBS) and blocked with normal rabbit serum. Slides were then incubated for 1 h in a 1 : 10 dilution of anti-pan-cytokeratin (5/6/8/17/19)-FITC (MNF116, Dako, Cambridge, UK) in TBP. The nuclei were then counterstained with 20 *μ*g ml^−1^ propidium iodide (PI; Sigma-Aldrich Company Ltd, Poole, UK) and 100 *μ*g ml^−1^ RNase (Sigma-Aldrich Company Ltd, Poole, UK) for 30 min. Slides were mounted in Vectashield containing 5 *μ*g ml^−1^ PI (Vector Laboratories Ltd, Peterborough, UK). The MCF7 breast cancer cell line was used as a positive control, and an isotype mouse IgG1 antibody as a negative control.

Slides were analysed using a Laser Scanning Cytometer (CompuCyte Inc., Cambridge, MA, USA) with WinCyte PC-based software as we have described previously (Zabaglo *et al*, 2003). Briefly, the slides were scanned using the argon laser 488-nm line, and the cells separated on the basis of the DNA fluorescence area, maximal pixel intensity, integrated fluorescence and perimeter into cell clumps, doublets and single cells. Green fluorescence (FITC-labelled cytokeratin) against integrated red fluorescence was displayed for each of the three cell populations and cytokeratin-positive cells identified. After analysis, all the cytokeratin-positive cells were relocated and examined by eye; only cells fulfilling the criteria of the European ISHAGE Working Group (European ISHAGE Working Group, 1999) were accepted as positive.

*Cell filtration followed by LSC*: Blood samples, diluted 1 : 1 in PBS 2 mM EDTA, were filtered under gravity through a Poretics polycarbonate Track-Etch-type (PCTE) membrane (Genetic Research Instrumentation Ltd, Braintree, UK) with calibrated 8 *μ*m cylindrical pores, as described previously (Zabaglo *et al*, 2003). The filters were then washed with PBS and fixed in 100% methanol prior to being removed from their holders and attached to a microscope slide. Slides were stained for cytokeratin and LSC analysis performed as for samples prepared by IMS.

*Real-time RT–PCR*: Samples were subjected to density gradient enrichment using Histopaque-1077 (Sigma-Aldrich Company Ltd, Poole, UK) as described for IMS. The cell pellets were stored in liquid nitrogen, and RNA extraction performed using TRIzol Reagent (Invitrogen Ltd, Paisley, UK) and RNA quantification using an Agilent 2100 Bioanalyser (Agilent Technologies Ltd, West Lothian, UK). DNase treatment was carried out using an RQ1 RNase-free DNase kit (Promega, Southampton, UK), according to the manufacturer's instructions. The RNA pellets were dissolved in nuclease-free water and stored at −70°C prior to use.

Primers and probes were designed for CK19, mammaglobin, PIP and the ribosomal RNA RPL19 using Primer Express™ software (Applied Biosystems, Warrington, UK). Where possible, primers and probes were designed to span intron–exon junctions, and in the case of CK19 to capitalise on sequences of nonhomology between CK19 cDNA and its known pseudogenes (Ruud *et al*, 1999). BLAST sequence similarity searches (www.ncbi.nlm.nih.gov/BLAST) were performed to confirm that there was no significant homology between the sequences chosen and other genes in the database. The final sequences and labels used are shown in Table 1 . The oligonucleotides were synthesised by Applied Biosystems (Warrington, UK).

Reverse transcription and real-time RT–PCR was carried out using the ABI Prism 7900HT operating system (Applied Biosystems, Warrington, UK). For each test gene, 10 *μ*l of DNase-treated RNA (20 ng *μ*l^−1^) was added to 50 *μ*l of 2 × AmpliTaq Gold® DNA polymerase master mix, 2.5 *μ*l 40 × Multiscribe™ reverse transcriptase (both Applied Biosystems, Warrington, UK), forward and reverse primers and probe at the optimised concentrations, and nuclease-free water to a total volume of 100 *μ*l. A measure of 25 *μ*l of this mix was added to three wells such that each sample was run in triplicate with 50 ng of RNA template. All sample plates were run with positive controls (T47D breast cancer cell line RNA) and no-template negative controls. The following thermal cycling conditions were used: 48°C for 30 min; 95°C for 10 min; 40 cycles of 95°C for 15 s followed by 60°C for 60 s.

Mean threshold (*C*_t_) values, the number of cycles after which the emitted fluorescence crossed the threshold fluorescence, were calculated for each sample for each of the test genes and the RPL19 normaliser. These were converted to mean normalised gene expression (MNE) using the following equation (Muller *et al*, 2002): 
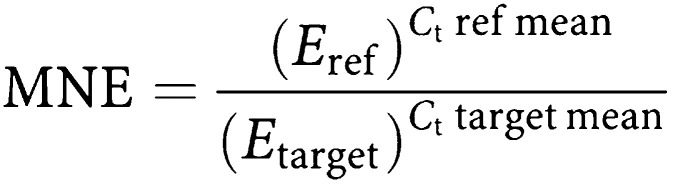
 where MNE is the mean normalised expression, *E*_ref_ is the PCR amplification efficiency of the reference gene, *E*_target_ is the PCR amplification efficiency of the target gene, *C*_t_ ref is the threshold cycle of the PCR amplification of the reference gene and *C*_t_ target is the threshold cycle of PCR amplification of the target gene. When the PCR amplification curve had not passed the threshold fluorescence after 40 cycles, the marker was described as undetectable in that sample and the MNE designated zero.

### Statistics

Differences between the number of circulating cell numbers between the control and patient populations were assessed using the Mann–Whitney *U*-test for unpaired non-normally distributed groups. Differences in cell numbers between the two cytometric techniques were analysed with the Wilcoxon signed-rank test for paired non-normally distributed groups. Correlations between the three techniques were assessed by calculating Spearman's rank correlation coefficient. Differences in positivity rates between the three techniques were assessed using the McNemar test. The *χ*^2^ test was used to assess the relation between patient characteristics and rates of positive samples. Values of *P*<0.05 were considered statistically significant. StatView 4.5 software (Cherwell Scientific, UK) was used throughout.

## RESULTS

### Baseline characteristics

Blood samples were taken from 40 patients with metastatic breast cancer and from 20 healthy volunteers. All analyses were performed blinded to the patient/control status of samples. The patients' baseline characteristics are shown in Table 2 .

## DETECTION BY LSC FOLLOWING IMS

**Figure 1:**
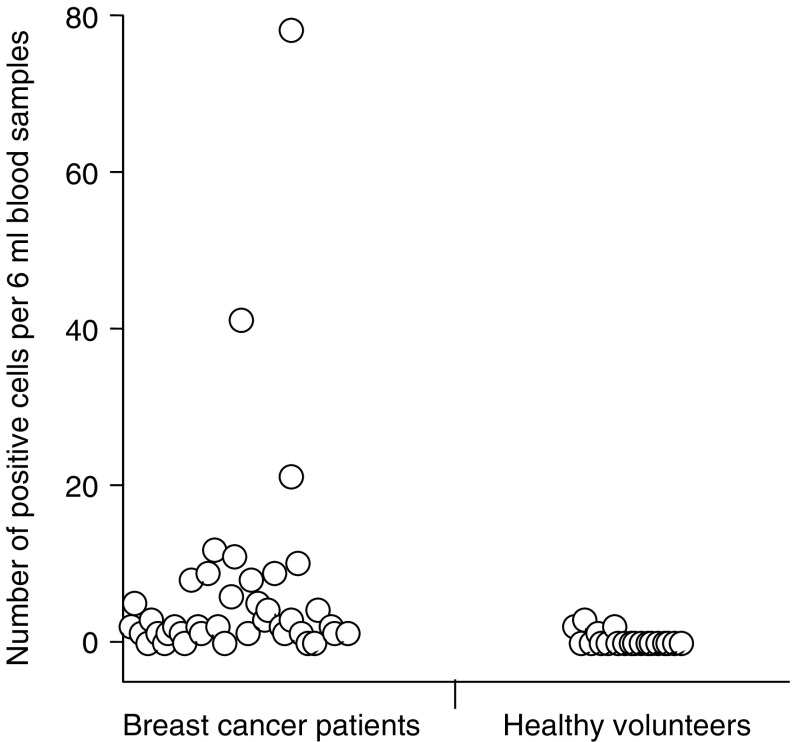
Number of positive epithelial cells detected per 6 ml blood sample in breast cancer patients and healthy volunteers when blood samples were analysed by LSC following IMS.

In the 20 control subjects, the mean number of positive epithelial cells detected was 0.40 6 ml^−1^ using LSC following IMS. In the 40 patients with metastatic breast cancer, the mean number of positive cells detected was 6.55 6 ml^−1^ (*P*<0.001, Mann–Whitney *U*-test). Positive cells were identified in the blood of four out of 20 (20%) healthy volunteers compared with 34 out of 40 (85%) patients with metastatic breast cancer (Figure 1). In common with a previous study, the upper 95% confidence interval of mean cell numbers in controls was used as a cutoff to define positive samples in patients with metastatic breast cancer (Aerts *et al*, 2001). Using such a threshold, 19 out of 40 (48%) samples from patients with metastatic breast cancer were positive. An alternative way to analyse this data is to set a threshold value for positive samples in the patient population, which corresponds to 100% specificity in the control population. Using this approach 15 out of 40 (38%) samples from patients with metastatic breast cancer were positive.

### Detection by LSC following cell filtration

**Figure 2:**
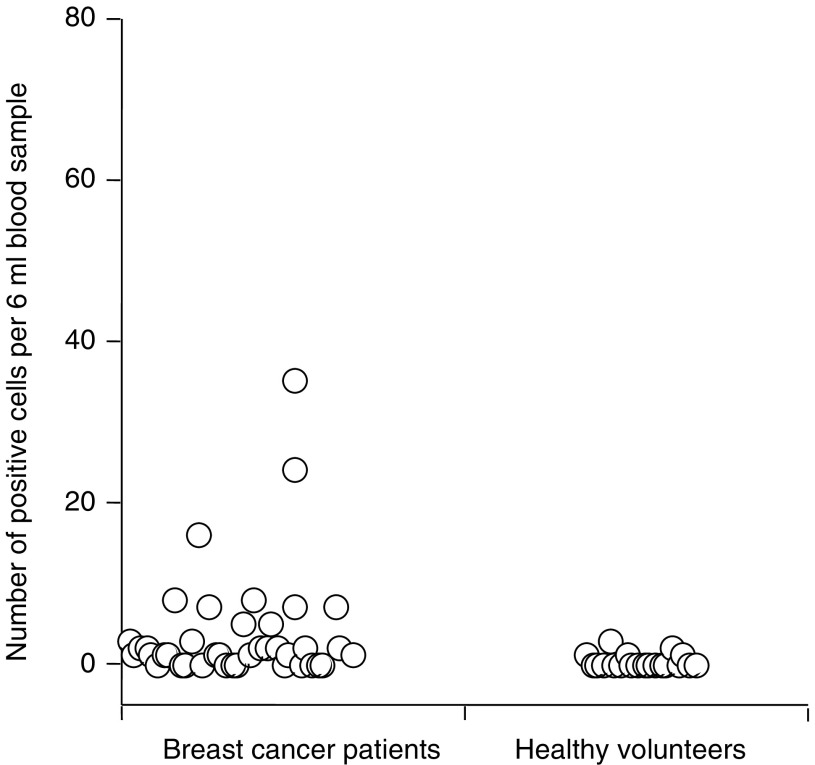
Number of positive epithelial cells detected per 6 ml blood sample in breast cancer patients and healthy volunteers when blood samples were analysed by LSC following cell filtration enrichment.

In the 20 control subjects, the mean number of positive epithelial cells detected was 0.42 6 ml^−1^ blood sample using LSC following cell filtration. In the 40 patients with metastatic breast cancer, the mean number of positive cells detected was 3.87 6 ml^−1^ (*P*=0.0012, Mann–Whitney *U*-test). Positive cells were identified in the blood of five out of 20 (25%) healthy volunteers compared with 28 out of 40 (70%) patients with metastatic breast cancer (Figure 2). Using the upper 95% confidence interval of mean cell numbers in controls as a cutoff, 12 out of 40 (30%) samples from patients with metastatic breast cancer were positive. Alternatively ensuring 100% specificity in the control population, 10 out of 40 (25%) samples from patients with metastatic breast cancer were positive.

### Detection by real-time RT–PCR for CK19, mammaglobin and PIP

**Figure 3:**
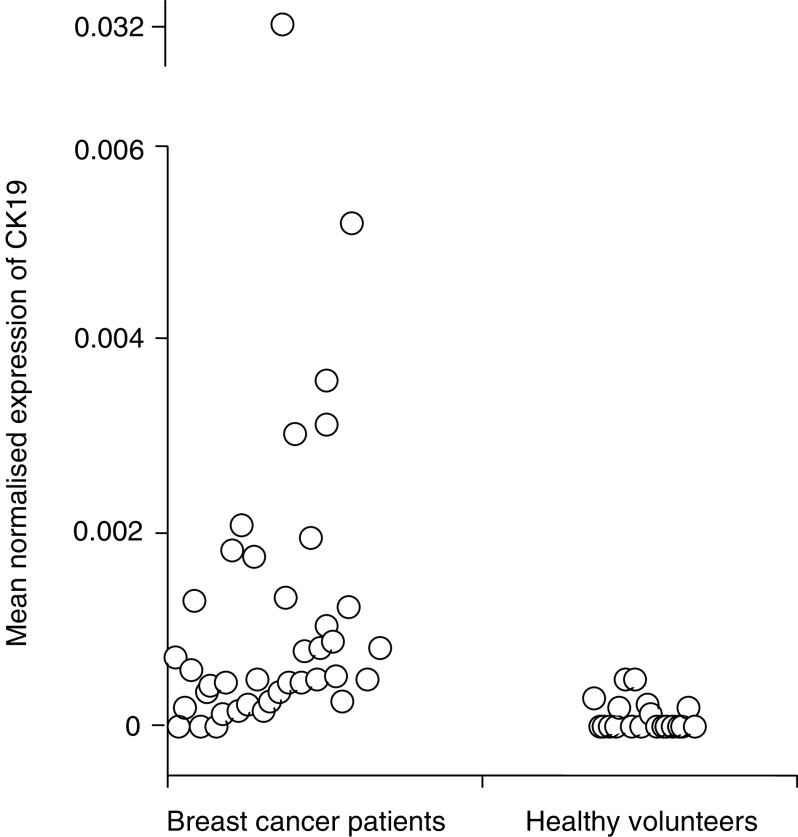
Mean normalised expression of CK19 detected in blood samples from patients with metastatic breast cancer and healthy volunteers when samples were analysed by real-time RT–PCR (CK19, cytokeratin 19).

**Figure 4:**
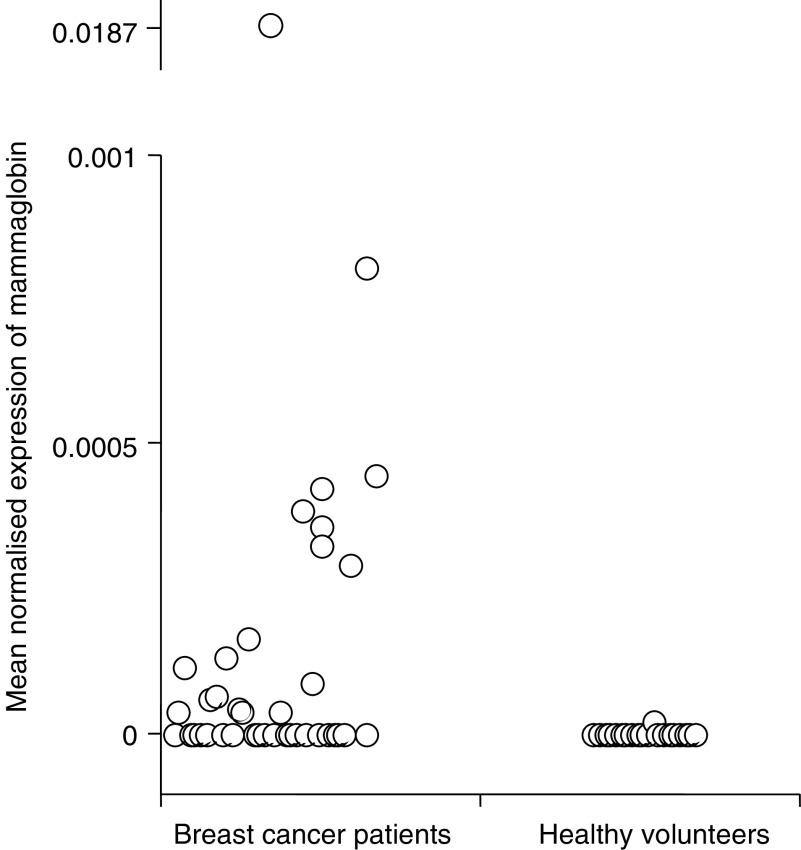
Mean normalised expression of mammaglobin detected in blood samples from patients with metastatic breast cancer and healthy volunteers when samples were analysed by real-time RT–PCR.

**Figure 5:**
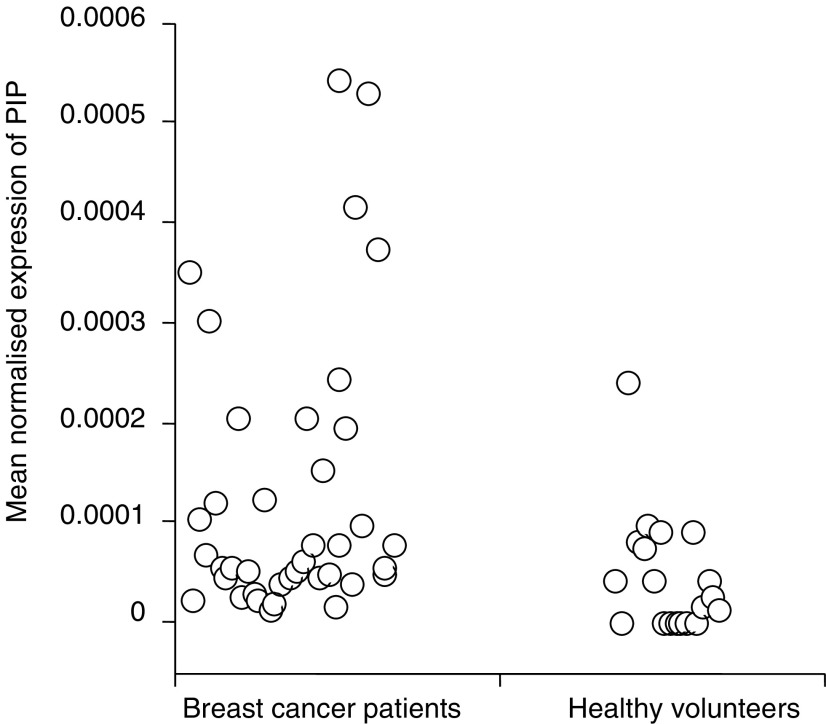
Mean normalised expression of PIP detected in blood samples from patients with metastatic breast cancer and healthy volunteers when samples were analysed by real time RT-PCR. (PIP, prolactin-inducible peptide).

The MNE values for the patients and healthy volunteer controls for the three PCR targets are shown in Table 3 . Differences in MNE between the healthy volunteers and patients were statistically significant for all three markers: CK19 (*P*<0.0001), mammaglobin (*P*=0.0011) and PIP (*P*=0.002). There were no statistically significant differences between the *C*_t_ values for RPL19 for the patients and controls (22.53 *vs* 22.47, *P*=0.58). The MNE for individual subjects for each of the three markers are shown in Figures 3, 4 and 5.

The upper 95% confidence interval of MNE in control subjects was used as a cutoff to define positive samples in patients with metastatic breast cancer. Using this threshold, 24 out of 40 (60%) samples from patients with metastatic breast cancer were positive for CK19, 18 out of 40 (45%) were positive for mammaglobin and 14 out of 40 (35%) were positive for PIP. Using a threshold to ensure 100% specificity in the control population 22 out of 40 (55%) samples from patients with metastatic breast cancer were positive for CK19 compared with 18 out of 40 (45%) for mammaglobin and seven out of 40 (18%) for PIP.

Regarding all 40 blood samples from the patients and using the 95% confidence interval threshold: seven (18%) were negative for all of the genes, 16 (39%) positive for only one gene, 11 (28%) positive for two genes and six (15%) positive for all three genes. In the 20 healthy volunteers: two (10%) were positive for one gene, but none of them was positive for more than one gene (Table 4 ). Using a threshold to ensure 100% specificity in the control population, 11 (28%) of patient samples were negative for all of the genes, 15 (38%) positive for only one gene, 10 (25%) positive for two genes and four (10%) positive for all three genes (Table 4).

There were no significant differences between the number of samples positive for CK19 and mammaglobin, and PIP and mammaglobin. However, significantly more samples from patients with metastatic breast cancer were positive for CK19 than for PIP (60 *vs* 35%, *P*=0.014). Concordant samples were defined as those where the patient sample was reported as either positive or negative by both of the markers being used. As such, the concordance between CK19 and mammaglobin was 65%, between CK19 and PIP was 53%, and between PIP and mammaglobin was 50%.

### Comparisons between the three techniques

The differences in mean cell numbers detected by LSC following IMS and cell filtration in patients with metastatic breast cancer did not reach statistical significance (Wilcoxon's signed rank test, *P*=0.08). There was also no significant difference between the number of samples defined as positive by LSC following IMS and cell filtration (48 *vs* 30%, *P*=0.18, McNemar test). There were significant correlations between the numbers of cells detected by LSC following cell filtration and IMS both in patients with metastatic breast cancer and in controls (Spearman's rank correlation coefficient, *ρ*=0.432, *P*=0.007 and *ρ*=0.742, *P*=0.0012, respectively).

Using the 95% confidence interval threshold in controls to define positive samples, patients with metastatic breast cancer were significantly more likely to be positive using the real-time RT–PCR assay for CK19 than using cell filtration followed by LSC (60 *vs* 30%, *P*=0.006, McNemar test), but this was not the case comparing the CK19 RT–PCR assay with IMS followed by LSC (60 *vs* 48%, *P*=0.06). However, when all three PCR markers were used, samples from patients with metastatic breast cancer were significantly more likely to be positive for at least one of the markers than they were likely to be positive by cell filtration or IMS followed by LSC (83 *vs* 30%, *P*<0.001 and 83 *vs* 48%, *P*<0.001, respectively).

Using the threshold set at 100% specificity, samples from patients with metastatic breast cancer were also significantly more likely to be positive for at least one of the markers than to be positive by cell filtration or IMS followed by LSC (73 *vs* 25%, *P*<0.001 and 73 *vs* 38%, *P*<0.01, respectively).

Concordant samples were defined as those where the sample from a patient was reported as either positive or negative by both of the techniques being compared. As a result, the concordance between samples analysed by cell filtration and IMS was 65%. Of the 12 patients positive by cell filtration, nine were also positive by IMS. When IMS and real-time RT–PCR for CK19, mammaglobin and PIP were compared, the concordances were 65, 70 and 50%, respectively. The corresponding figures for cell filtration compared with the real-time RT–PCR assays were: 60, 60 and 50%.

The concordance between samples defined as positive as a result of expressing all three test genes and those defined as positive by cell filtration and IMS was 73 and 60%, respectively.

## DISCUSSION

The detection of circulating tumour cells has considerable potential to influence the management of patients with breast cancer. However, using existing techniques there is considerable variability in the rates of positive samples in patients with metastatic breast cancer and in control populations. Therefore, it is important to compare directly techniques, in order to ascertain the optimal approaches to use.

Using LSC following IMS and cell filtration more circulating epithelial cells were detected in the blood of patients with metastatic breast cancer than in control healthy volunteers. Similarly, the expression of CK19, mammaglobin and PIP was found to be significantly higher in the blood of patients with metastatic breast cancer than in controls. A cutoff for positivity was defined for all three techniques based on the upper 95% confidence intervals of mean cell numbers in the control population. On this basis, 30% of the patient samples were positive by filtration, 48% by IMS enrichment and 35–60% using real-time RT–PCR depending on the test gene used. The rates of positive samples using the cytometric techniques and real-time RT–PCR are within the previously observed ranges using such techniques to detect circulating epithelial cells in the blood of patients with metastatic breast cancer (Smith *et al*, 2000; Bosma *et al*, 2002; Baker *et al*, 2003; Stathopoulou *et al*, 2003; Ring *et al*, 2004).

This study demonstrates the benefit of using more than one target for RT–PCR amplification. If using CK19, mammaglobin or PIP alone, then 60, 45 and 35% of samples would be expected to be positive. However, 82% of patients were positive for at least one of these markers. The relatively low concordance values for positivity between the different markers (50–65%) probably reflects not only differences in sensitivity but also that the different markers are identifying different populations of cells: in fact, of the 16 CK19-negative patients, five were PIP positive. This reinforces the benefit of using a multiple marker PCR assay. Similar results were reported by Taback and colleagues who used RT–PCR to assess the expression of four genes in the blood of 65 patients with breast cancer (stage I–IV) and 40 normal volunteers (Taback *et al*, 2001). Individual markers were detected in 11–37% of patients, but at least one marker was detected in the blood of 69% of patients. These two studies demonstrate that although no one marker may be ideal for the detection of circulating tumour cells, the use of a multiple marker assay may significantly improve sensitivity.

In a further study published by Baker *et al*, 13 out of 20 (65%) patients with metastatic breast cancer had overexpression in their blood of at least one of six test genes (Baker *et al*, 2003). This study also provides evidence that enrichment using porous barrier density gradient centrifugation reduces background expression of CK19 and MUC1 compared with a Ficoll density gradient similar to that used in the current study. Therefore, it may prove to be possible to further improve on the real-time RT–PCR results reported here by modifying the enrichment step.

As far as comparisons between the techniques are concerned, the CK19 RT–PCR assay was found to be more sensitive than cell filtration followed by LSC, although there was no significant difference compared with IMS followed by LSC. However, when all three markers are taken into consideration, samples from patients with breast cancer are more likely to have a positive result with at least one of the markers than either of the two cytometric techniques (both *P*<0.001). These results show that when a multiple marker real-time RT–PCR assay is used, its sensitivity exceeds that of both an established and a novel cytometric technique. Previous studies using nonquantitative RT–PCR also reveal more positive results when patient samples are analysed by PCR than by immunocytochemical techniques (Schoenfeld *et al*, 1997; Lambrechts *et al*, 1999). However, these results may be compromised by lower specificity (Lambrechts *et al*, 1999). Slade and colleagues used a competitive PCR reaction to enable relative quantification of samples and to establish a cutoff point for positivity based on analyses of the blood of normal individuals (Slade *et al*, 1999). Using such an approach for CK19, it was found that 50% of samples from patients with metastatic breast cancer were positive by PCR compared with 42% of samples analysed using immunocytochemistry (Smith *et al*, 2000).

Aside from the relative sensitivities and specificities of the different techniques, the correlations between the techniques and their concordance are also of interest. Good correlations were observed between cell numbers detected by the two cytometric techniques and between real-time RT–PCR for CK19 and IMS enrichment. However, when a cutoff for positivity is established, the concordance between techniques was between 68 and 75%, meaning that although the sensitivity of techniques may be similar, the samples being called positive and negative are not necessarily the same. Other studies show qualitative concordance between PCR-based assays and immunocytochemistry of 71–77% (Schoenfeld *et al*, 1997; Smith *et al*, 2000). In the case of the cytometric techniques, the limited concordance may reflect the differences in specimen handling. The differences between the real-time RT–PCR technique and the cytometric techniques may be further explained by the different markers detected and the determination of expression at an RNA as opposed to a protein level. An important corollary of this observation is that cytokeratin-positive cells identified by one technique should not be assumed to have the same significance as those detected by another technique: the populations of cells detected may be distinct and have different clinical, biological and prognostic significance.

The quantitative nature of the real-time RT–PCR assay makes it an ideal technique by which to establish a normal range of expression for markers in controls. This was a comparative study and hence the same approach was used in the cytometric techniques. Such an approach predetermines the specificity of the assay. In order to assess fully the sensitivity and specificity of these techniques, the cohort presented in this study should be regarded as a training set. The established cutoffs for positivity should now be used in an independent validation set, to establish fully the clinical performance of the technique. A second issue when considering RNA-based assays such as RT–PCR is that RNA is inherently labile and therefore blood should be analysed as soon as possible after sampling. This may not be practical if samples (as seems likely) need to be transferred to a central laboratory for analysis. This practical issue may limit the widespread clinical utility of the PCR approach, although recently blood RNA tubes have become available (PAXgene™, Qiagen Ltd, Crawley, UK), which may be able to overcome this problem.

These caveats aside, these data show that using a real-time RT–PCR assay there is evidence of circulating tumour cell dissemination in the blood of up to 80% of patients with metastatic breast cancer. Although such analysis of blood samples may enable the monitoring of responses to treatment, in the absence of further characterisation of the significance of the cells, the clinical utility of the approach may be limited. The ascertainment of circulating individual cell phenotype at relapse and during therapy could significantly enhance our understanding of mechanisms of resistance and facilitate targeting of therapy, but is only likely to be possible using cytometric techniques. Circulating tumour cell detection theoretically would have its greatest impact in the management of early breast cancer, where risk stratification may be improved and responses to adjuvant therapy and the detection of relapse monitored. However, not all patients even with metastatic disease have detectable circulating tumour cells, so whether a significant role will emerge will depend on applying such techniques in large prospective studies in patients with early breast cancer.

In summary, the data presented suggest that the use of a multiple marker real-time RT–PCR assay may be a more sensitive technique by which to detect circulating breast cancer cells than the use of a novel or an established cytometric technique. This does not necessarily mean that the PCR-based assay is superior to the other techniques, as different information may be acquired by the different approaches. If the aim of the technique is to assess blood ‘tumour burden’, such as if one is monitoring responses to treatment, then real-time RT–PCR may be the superior approach. However, if it is desirable to visualise cells directly and perhaps perform further phenotypic or molecular characterisation, then a cytometric approach may be preferable.

## Figures and Tables

**Table 1:** Primer and probe sequences for the target genes

**Target gene**	**Gene bank accession number**	**Forward primer sequence**	**Reverse primer sequence**	**Probe sequence**	**5′ label**	**3′ quencher**
CK19	NM002276	TGCGGGACAAGATTCTTGGT	TCTCAAACTTGGTTCGGAAGTCA	ACCATTGAGAACTCCAGGATTG TCCTGCA	6-FAM	TAMRA
Mammaglobin	AF015224	TGCCATAGATGAATTGA AGGAATG	TCATATATTAATTGCATAAACA CCTCAACA	ACCAAACGGATGAAACT	6-FAM	MGB
PIP	J03460	TGGAAGCCCTGTCTGTTTGC	AGCAGAAATTCCAGCCAAGTTTC	CCCAGGTGATTTCC	6-FAM	MGB
RPL19	NM000981	CCATGAGTATGCTCAGGCTTCA	CTGACGGGAGTTGGCATTG	CCTCTAGTGTCCTCCGC TGTGGCAAG	VIC™	TAMRA

CK19=cytokeratin 19; PIP=prolactin-inducible peptide; 6-FAM, 6-carboxyfluorescein; TAMRA, 6-carboxytetramethylrhodamine; MGB, minor groove binder, nonfluorescent quencher.

**Table 2:** Baseline characteristics of the 40 patients with metastatic breast cancer enrolled in the study

**Characteristic**	**Patients with stage IV breast cancer (*n*=40)**
Median age (years) (range)	56.5 (24–77)

*Sites of metastatic disease*	
Bone	23 (58%)
Lymph node	22 (55%)
Liver	24 (60%)
Lung/pleural	21 (53%)
Skin	5 (13%)

*Number of sites of disease*	
1	7 (18%)
2	17 (43%)
>2	16 (40%)

*Current therapy*	
Endocrine	22 (55%)
Chemotherapy	0
None	18 (45%)

*Number of previous chemotherapy regimens*	
0	5 (13%)
1	19 (48%)
2	10 (25%)
>2	6 (15%)

*Number of previous endocrine regimens*	
0	7 (18%)
1	14 (35%)
2	12 (30%)
>2	7 (18%)

Previous radiation therapy for metastatic disease	16 (40%)

**Table 3:** MNE of CK19, mammaglobin and PIP assessed in the blood of 40 patients with metastatic breast cancer and 20 healthy volunteers by real-time RT–PCR

**Test gene**	**MNE healthy volunteers (*n*=20) (range)**	**Healthy volunteers in whom test gene detectable (*n*=20)**	**MNE patients with metastatic breast cancer (*n*=40) (range)**	**Patients in whom test gene detectable (*n*=40)**
CK19	0.0000996 (0–0.00049)	7 (35%)	0.001780 (0–0.032)	37 (93%)
Mammaglobin	0.0000013 (0.000025)	1 (5%)	0.000578 (0–0.01870)	18 (45%)
PIP	0.000043 (0–0.000241)	13 (65%)	0.000128 (0.000014-0.000543)	40 (100%)

MNE=mean normalised expression; CK19=cytokeratin 19; PIP=prolactin-inducible peptide; RT–PCR=reverse transcriptase–polymerase chain reaction.

**Table 4:** Number of healthy volunteers and patients positive for none, at least one or all three of the test genes (CK19, mammaglobin and PIP) by real-time RT–PCR

	**Healthy volunteers (*n*=20)**		**Patients (*n*=40)**	
	**Level of specificity**		**Level of Specificity**	
**Number of genes positive**	**95%**	**100%**	**95%**	**100%**
None	18 (90%)	20 (100%)	7 (18%)	11 (28%)
⩾1	2 (10%)	0 (0%)	33 (83%)	29 (73%)
All 3	0 (0%)	0 (0%)	6 (15%)	4 (10%)

CK19=cytokeratin 19; PIP=prolactin-inducible peptide; RT–PCR=reverse transcriptase–polymerase chain reaction.

Positive samples defined using either the upper 95% confidence interval of levels in the healthy volunteers to describe positive samples or using a threshold that provided 100% specificity in healthy volunteers.

## References

[bib1] Aerts J, Wynendaele W, Paridaens R, Christiaens MR, van den Bogaert W, van Oosterom AT, Vandekerckhove F (2001) A real-time quantitative reverse transcriptase polymerase chain reaction (RT–PCR) to detect breast carcinoma cells in peripheral blood. Ann Oncol 12: 39–46 11249047 10.1023/a:1008317512253

[bib2] Baker MK, Mikhitarian K, Osta W, Callahan K, Hoda R, Brescia F, Kneuper-Hall R, Mitas M, Cole DJ, Gillanders WE (2003) Molecular detection of breast cancer cells in the peripheral blood of advanced-stage breast cancer patients using multimarker real-time reverse transcription–polymerase chain reaction and a novel porous barrier density gradient centrifugation technology. Clin Cancer Res 9: 4865–4871 14581359

[bib3] Bartek J, Bartkova J, Schneider J, Taylor-Papadimitriou J, Kovarik J, Rejthar A (1986) Expression of monoclonal antibody-defined epitopes of keratin 19 in human tumours and cultured cells. Eur J Cancer Clin Oncol 22: 1441–1452 2439341 10.1016/0277-5379(86)90077-5

[bib4] Bosma AJ, Weigelt B, Lambrechts AC, Verhagen OJ, Pruntel R, Hart AAM, Rodenhuis S, van't Veer LJ (2002) Detection of circulating breast tumor cells by differential expression of marker genes. Clin Cancer Res 8: 1871–1877 12060630

[bib5] Brandt B, Roetger A, Heidl S, Jackisch C, Lelle RJ, Assmann G, Zanker KS (1998) Isolation of blood-borne epithelium-derived c-erbB-2 oncoprotein-positive clustered cells from the peripheral blood of breast cancer patients. Int J Cancer 76: 824–828 9626348 10.1002/(sici)1097-0215(19980610)76:6<824::aid-ijc10>3.0.co;2-2

[bib6] Clark JW, Snell L, Shiu RP, Orr FW, Maitre N, Vary CPH, Cole DJ, Watson PH (1999) The potential role for prolactin-inducible protein (PIP) as a marker of human breast cancer micrometastasis. Br J Cancer 81: 1002–1008 10576657 10.1038/sj.bjc.6690799 PMC2362951

[bib7] European ISHAGE Working Group for the Standardisation of Tumour Cell Detection (1999) Standardisation of the immunocytochemical detection of cancer cells in BM and blood: I. Establishment of objective criteria for the evaluation of immunostained cells. Cytotherapy 1: 377–388 20426539 10.1080/0032472031000141283

[bib8] Lambrechts AC, Bosma AJ, Klaver SG, Top B, Perebolte L, van't Veer LJ, Rodenhuis S (1999) Comparison of immunocytochemistry, reverse transcriptase polymerase chain reaction, and nucleic acid sequence-based amplification for the detection of circulating breast cancer cells. Breast Cancer Res Treat 56: 219–231 10573113 10.1023/a:1006261731125

[bib9] Mitas M, Mikhitarian K, Almeida JS, Gillanders WE, Cole DJ (2003) Application of population-based statistical theory to a multi-institutional prospective cohort study indicates that mammaglobin is the most reliable marker for the detection of micrometastatic cancer. San Antonio Breast Cancer Symposium abstract 1018

[bib10] Moll R (1994) Cytokeratins in the histological diagnosis of malignant tumors. Int J Biol Markers 9: 63–69 7523543 10.1177/172460089400900201

[bib11] Muller PY, Janovjak H, Miserez AR, Dobbie Z (2002) Processing of gene expression data generated by quantitative real-time RT–PCR. Biotechniques 32: 1372–1374, 1376, 1378–1379 12074169

[bib12] Pachmann K, Heiss P, Demel U, Tilz G (2001) Detection and quantification of small numbers of circulating tumour cells in peripheral blood using laser scanning cytometer (LSC). Clin Chem Lab Med 39: 811–817 11601678 10.1515/CCLM.2001.134

[bib13] Racila E, Euhus D, Weiss AJ, Rao C, McConnell J, Terstappen LW, Uhr JW (1998) Detection and characterization of carcinoma cells in the blood. Proc Natl Acad Sci USA 95: 4589–4594 9539782 10.1073/pnas.95.8.4589 PMC22534

[bib14] Ring A, Smith IE, Dowsett M (2004) Circulating tumour cells in breast cancer. Lancet Oncol 5: 79–88 14761811 10.1016/S1470-2045(04)01381-6

[bib15] Ruud P, Fodstad O, Hovig E (1999) Identification of a novel cytokeratin 19 pseudogene that may interfere with reverse transcriptase–polymerase chain reaction assays used to detect micrometastatic tumor cells. Int J Cancer 80: 119–125 9935241 10.1002/(sici)1097-0215(19990105)80:1<119::aid-ijc22>3.0.co;2-x

[bib16] Schoenfeld A, Kruger KH, Gomm J, Sinnett HD, Gazet JC, Sacks N, Bender HG, Luqmani Y, Coombes RC (1997) The detection of micrometastases in the peripheral blood and bone marrow of patients with breast cancer using immunohistochemistry and reverse transcriptase polymerase chain reaction for keratin 19. Eur J Cancer 33: 854–861 9291805 10.1016/s0959-8049(97)00014-2

[bib17] Silva AL, Tome MJ, Correia AE, Passos-Coelho JL (2002) Human mammaglobin RT–PCR assay for detection of occult breast cancer cells in hematopoietic products. Ann Oncol 13: 422–429 11996474 10.1093/annonc/mdf107

[bib18] Singletary SE, Allred C, Ashley P, Bassett LW, Berry D, Bland KI, Borgen PI, Clark GM, Edge SB, Hayes DF, Hughes LL, Hutter RV, Morrow M, Page DL, Recht A, Theriault RL, Thor A, Weaver DL, Wieand HS, Greene FL (2002) Revision of the American Joint Committee on Cancer staging system for breast cancer. J Clin Oncol 20: 3628–3636 12202663 10.1200/JCO.2002.02.026

[bib19] Slade MJ, Smith BM, Sinnett HD, Cross NC, Coombes RC (1999) Quantitative polymerase chain reaction for the detection of micrometastases in patients with breast cancer. J Clin Oncol 17: 870–879 10071278 10.1200/JCO.1999.17.3.870

[bib20] Smith BM, Slade MJ, English J, Graham H, Luchtenborg M, Sinnett HD, Cross NCP, Coombes RC (2000) Response of circulating tumor cells to systemic therapy in patients with metastatic breast cancer: comparison of quantitative polymerase chain reaction and immunocytochemical techniques. J Clin Oncol 18: 1432–1439 10735890 10.1200/JCO.2000.18.7.1432

[bib21] Stathopoulou A, Gizi A, Perraki M, Apostolaki S, Malamos N, Mavroudis D, Georgoulias V, Lianidou ES (2003) Real-time quantification of CK-19 mRNA-positive cells in peripheral blood of breast cancer patients using the LightCycler system. Clin Cancer Res 9: 5145–5151 14613993

[bib22] Stathopoulou A, Vlachonikolis I, Mavroudis D, Perraki M, Kouroussis C, Apostolaki S, Malamos N, Kakolyris S, Kostakis A, Xenidis N, Reppa D, Georgoulias V (2002) Molecular detection of cytokeratin-19-positive cells in the peripheral blood of patients with operable breast cancer: evaluation of their prognostic significance. J Clin Oncol 20: 3404–3412 12177100 10.1200/JCO.2002.08.135

[bib23] Taback B, Chan AD, Kuo CT, Bostick PJ, Wang HJ, Giuliano AE, Hoon DS (2001) Detection of occult metastatic breast cancer cells in blood by a multimolecular marker assay: correlation with clinical stage of disease. Cancer Res 61: 8845–8850 11751407

[bib24] Vona G, Sabile A, Louha M, Sitruk V, Romana S, Schutze K, Capron F, Franco D, Pazzagli M, Vekemans M, Lacour B, Brechot C, Paterlini-Brechot P (2000) Isolation by size of epithelial tumor cells: a new method for the immunomorphological and molecular characterization of circulating tumor cells. Am J Pathol 156: 57–63 10623654 10.1016/S0002-9440(10)64706-2 PMC1868645

[bib25] Witzig TE, Bossy B, Kimlinger T, Roche PC, Ingle JN, Grant C, Donohue J, Suman VJ, Harrington D, Tuerro-Bueno J, Kauer KD (2002) Detection of circulating cytokeratin-positive cells in the blood of breast cancer patients using immunomagnetic enrichment and digital microscopy. Clin Cancer Res 8: 1085–1091 12006523

[bib26] Zabaglo L, Ormerod MG, Parton M, Ring A, Smith IE, Dowsett M (2003) Cell filtration-laser scanning cytometry for the characterisation of circulating breast cancer cells. Cytometry 55A: 102–108 10.1002/cyto.a.10071 14505315

[bib27] Zach O, Kasparu H, Krieger O, Hehenwarter W, Girschikofsky M, Lutz D (1999) Detection of circulating mammary carcinoma cells in the peripheral blood of breast cancer patients via a nested reverse transcriptase polymerase chain reaction assay for mammaglobin mRNA. J Clin Oncol 17: 2015–2019 10561252 10.1200/JCO.1999.17.7.2015

